# Weight and waist-to-hip ratio change pattern during the first five years of survival: data from a longitudinal observational Chinese breast cancer cohort

**DOI:** 10.1186/s12885-021-08554-5

**Published:** 2021-07-20

**Authors:** Yuan-Yuan Lei, Suzanne C. Ho, Carol Kwok, Ashley Cheng, Ka Li Cheung, Roselle Lee, Frankie Mo, Winnie Yeo

**Affiliations:** 1 Department of Clinical Oncology, Prince of Wales Hospital, The Chinese University of Hong Kong, Shatin, New Territories, Hong Kong, SAR China; 2 Division of Epidemiology, the Jockey Club School of Public Health and Primary Care, the Chinese University of Hong Kong, New Territories, Hong Kong, SAR China; 3 Department of Clinical Oncology, Princess Margaret Hospital, Hong Kong, SAR China; 4 Hong Kong Cancer Institute, State Key Laboratory in Oncology in South China, Faculty of Medicine, the Chinese University of Hong Kong, New Territories, Hong Kong, SAR China

**Keywords:** Breast cancer, Body weight, Body mass index (BMI), Waist-to-hip ratio (WHR), Change, Chinese women

## Abstract

**Background:**

Body weight management was an important component in breast cancer survivorship care. The present study described the change patterns of body weight and waist-to-hip ratio (WHR) during the first 5 years of survival, and investigated potential factors associated with very substantial changes.

**Patients and methods:**

Based on a longitudinal cohort with 1462 Chinese women with breast cancer, anthropometric measurements including body weight, height, waist and hip circumferences were measured by trained interviewers following standard protocol at four time-points: baseline at study entry, 18-, 36- and 60-months follow up assessments (termed as T0, T1, T2 and T3, respectively). Body height was measured at baseline and body weight at cancer diagnosis were retrieved from medical record.

**Results:**

Compared to weight at breast cancer diagnosis, the median weight change was − 0.5 kg, 0 kg, + 0.5 kg, and + 1 kg at T0, T1, T2 and T3, respectively. During the first 5 years of survival, the proportion of women who were obese have slightly increased. At 60-months after diagnosis, only 14.3% of women had weight gain by > 5 kg; and the percentage of women who had weight gain by > 10% was 10.7%. Nearly half of patients had abdominal obesity at study entry, and this proportion were gradually increased to nearly 70% at 60-months follow-up. Multivariate analysis indicated that older age, and frequent sports participation during the first 5 years of survival were related to lower risk of very substantial weight gain (> 10%) at 60-month follow-up; patients aged 40–49 years, having ≥2 comorbidities and ER negative were associated with less likelihood of very substantial WHR substantial increase (> 10%) at 60-month follow-up.

**Conclusion:**

Weight gain was modest in Chinese breast cancer survivors during the first 5 years of survival, while central adiposity has become a contemporary public health issue. The incorporation of healthy weight and abdominal circumference patient education and management has a potential to improve cancer survivorship.

**Supplementary Information:**

The online version contains supplementary material available at 10.1186/s12885-021-08554-5.

## Introduction

Breast cancer is the most frequent cancer among female both in Western countries and in China [1, 2]. An analysis from 40 years of cancer registry data showed that the incidence of breast cancer have a tremendous increase, while the mortality rate only slightly increase in urban Chinese population [3]. It indicates that a rising number of women with breast cancer would live with the disease for longer time. In Hong Kong, breast cancer has also aroused heavy disease burden in local female [4]. Breast Cancer Survivorship Care Guideline established by the American Society of Clinical Oncology (ASCO) has underscored that adoption of a healthy lifestyle as an essential element of survivorship care [5]. For such, body weight management is an important component, and ASCO recommended that primary care clinicians should counsel breast cancer survivors to achieve and maintain a healthy body weight [5].

Weight gain averaging 1.5 to 2.1 kg during 1–2 year post-diagnosis has been very frequent in Caucasian women [6–9]. In Asian women, weight changes have also been observed after breast cancer diagnosis, but the results have been inconsistent, [10–13] with most of those studies being cross-sectional and involving small patient number [10, 12, 13]. The reasons for weight gain could be multifactorial. A series of factors have been suggested to be associated with weight gain, including chemotherapy, use of medication in association with chemotherapy such as dexamethasone, treatment-related amenorrhea, “stress eating” and reduced physical activity [13–16]. As weight management is an essential element in the long-term management of breast cancer survivors, there is still a need to fully investigate the pattern of weight change and explore potential risk factors associated with very substantial weight gain among Asian women with breast cancer.

The sole use of body mass index (BMI) cannot fully reflect fat distribution over body compartments [17]; therefore, waist-to-hip ratio (WHR), have been recommended for the measurement of abdominal obesity in recent decades [18]. Freedman et al. have reported that patients with breast cancer who had adjuvant chemotherapy would experience unfavorable changes in body composition without a significant weight change [19]. The pattern of WHR change after breast cancer diagnosis has not been fully illustrated. As previous study showed that ﻿Chinese and South Asian displayed a greater amount of visceral adipose tissue for a given waist circumference when compared to Europeans, the measurement of WHR in Asian may need special attention [20].

Based on longitudinal follow-up of a cohort of Chinese women with breast cancer, the present study has the following two aims: 1) to characterize the patterns of weight change from diagnosis to immediately post-diagnosis and at 18-, 36-, 60-months follow-up, and to identify potential socio-demographic, clinical and lifestyle factors associated with very substantial weight gain; 2) to describe the patterns of WHR change from immediately post-diagnosis to 18-, 36- and 60-months follow-up, and to identify potential factors associated with very substantial WHR increase.

## Patients and methods

### Study cohort

The Hong Kong NTEC-KWC Breast Cancer Survival Study (HKNKBCSS) was a prospective breast cancer cohort study, initiated to investigate the associations between lifestyle factors with breast cancer recurrence and mortality [21–25]. The inclusion criteria included patients of any age, had histologically confirmed breast cancer with American Joint Committee on Cancer (AJCC) stage 0-III diagnosed no more than 12 months before study entry, [26] female gender, mentally stable, Chinese ethnicity, able to read Chinese, and did not have prior history of breast or other cancers. Between January 2011 and February 2014, 1462 eligible patients provided written informed consent and participated in the study. The study was approved by the Joint CUHK-NTEC Clinical Research Ethics Committee and the KWC Research Ethics Committee of the Chinese University of Hong Kong and the Hong Kong Hospital Authority.

Consented patients were interviewed at four time-points: baseline at study entry (described as T0; conducted within 12-months after breast cancer diagnosis), 18-months follow-up (T1; conducted between 12 and 24 months after diagnosis), 36-months follow-up (T2; which was conducted between 30 and 42 months after diagnosis) and 60-months follow-up (T3; 54–66 months after diagnosis). A telephone call would be made prior to the planned interview, which would coincide with their scheduled clinic follow-up.

As of December 2017, the 60-months follow-up interview had been completed. Among 1462 patients who completed assessment at T0, 1310, 1162 and 1171 participants completed interviews at T1, T2 and T3, respectively (follow-up rate: 89.6, 79.5 and 80.1%, respectively). The present study was based on the anthropometric measurements data assessed during interviews at all four time-points.

### Data collection

During each follow-up interviews, patients were assessed with structured questionnaires conducted at baseline assessment, which collected socio-demographic characteristics (education level, marital status, working status and family income), reproductive history, menopausal status, active and passive smoking, alcohol use and prior medical history (self-reported comorbidities including but not limited to diabetes, hypertension, hyperlipidemia, chronic liver disease and chronic kidney disease). At T0 assessment, patients’ menopause status was classified as two groups: pre-menopausal and post-menopausal. Patients who had their last menstrual period within 1 year were regarded as pre-menopausal. Post-menopausal was defined as patients who had a cessation of menstruation for 12 months or longer. From T0 to T3 assessment, patients’ menopause status could be classified as three groups: pre-menopausal, peri-menopausal and post-menopausal. Peri-menopausal was defined as pre-menopausal patients at T0 who described a change in menopause status by T3.

Clinical information was retrieved by reviewing hospital medical records. ﻿These included patient’s age at breast cancer diagnosis, cancer characteristics [histology, AJCC stage, estrogen receptors (ER), progesterone receptors (PR) and human epidermal-growth-factor receptor 2 (HER2) status of the breast tumor] and treatment for breast cancer (type of breast surgery, details of adjuvant radiotherapy, chemotherapy and endocrine therapy).

Physical activity was measured by a validated modified Chinese Baecke questionnaire [27]. The MET code of each sport was based on the values in the Ainsworth compendium of physical activity [28]. According to the level of sports activity, patients were categorized into 3 groups as follow: never (0 MET-hours/week), rare/occasional (< 10 MET-hours/week) and frequent (≥10 MET-hours/week) physical activity. The cut-off point of 10 MET-hours/week was based on the recommendations for cancer survivors [29, 30]. Habitual dietary intake was collected by a validated and interviewer-administered food frequency questionnaire (FFQ) [31]. The average daily intake of nutrients, such as total energy, fat and other nutrients were calculated according to the Chinese Food Composition Table [32]. Meeting dietary recommendation on vegetables and fruits intake was defined as eating at least five servings (at least 400 g) of nonstarchy vegetables and fruits every day according to the WCRF/AICR guidelines for cancer survivors [29]. At T0 assessment, patients were asked to recall their habitual physical activity and dietary intake in the preceding 12 months before cancer diagnosis. At T1, T2 and T3 assessments, patients were asked to report these parameters over the previous 12 months.

### Anthropometric measurements

Patients who have cancer recurrence or died during the first five years did not measure their body weight in the subsequent follow-ups, and those patients did not include into the present analysis. Anthropometric measurements including body weight, height, waist and hip circumference were performed based on standard protocol. Trained interviewers measured body weight, waist and hip circumference at T0, T1, T2 and T3 assessment, respectively, and the body height was only measured at T0 assessment. Body height was measured to the nearest 0.1 cm with the patients in bare feet, back against the wall, heels together and eyes looking straight ahead. Body weight was measured to the nearest 0.1 kg with the participant in light clothing and bare feet using a TANITA Body Fat Scale (Model BF-522, TANITA, Japan). Weight at diagnosis was collected from hospital medical records.

Body mass index (BMI) was calculated by weight (kg) divided by the square of height (m^2^). According to BMI classification of the Asia-Pacific region, patients can be categorized into 5 groups as following: underweight < 18.5 kg/m^2^, normal 18.5–22.9 kg/m^2^, overweight 23–24.9 kg/m^2^, obese ≥25 kg/m^2^ [33]. Compared to weight at breast cancer diagnosis, absolute weight change at T0, T1, T2 and T3 assessment was calculated (weight at T0, T1, T2 or T3 - weight at diagnosis), and then classified into five groups: substantial loss (> 5 kg), moderate loss (> 2 kg and ≤ 5 kg), stable change (within 2 kg), moderate gain (> 2 kg and ≤ 5 kg) and substantial gain (> 5 kg). The relative percent of weight change at T0, T1, T2 and T3 assessment was also calculated (absolute weight change at T0, T1 or T2/weight at diagnosis*100), and then classified those changes into six groups: substantial loss (> 5%), moderate loss (> 2% and ≤ 5%), stable change (within 2%), moderate gain (> 2 and ≤ 5%), substantial gain (5–10%) and very substantial gain (> 10%).

WHR was calculated as the ratio of waist circumference to hip circumference, which is regarded as an index of abdominal obesity [34]. World Health Organization (WHO) expert consultation defines abdominal obesity as WHR above 0.85 for women [35]; patients were grouped into two categories: without abdominal obesity < 0.85 and with abdominal obesity ≥0.85. As WHR at diagnosis were not available, the value obtained at T0 assessment was used as reference in the measurement of post-diagnosis WHR change. Percentage of changes between T0 assessment and T1, T2 and T3 assessment were calculated (absolute WHR change at T1, T2 or T3/WHR at T0*100), and then classified those changes into six groups: substantial decrease (> 5%), moderate decrease (> 2% and ≤ 5%), stable change (within 2%), moderate increase (> 2% and ≤ 5%), substantial increase (5–10%) and very substantial increase (> 10%).

### Statistical analysis

Patients’ socio-demographic, clinical and lifestyle factors described as follows: continuous variables were expressed as means with standard deviation or median with range as appropriate, and categorical variables were ﻿summarized as patient number (n) and percentage (%). Univariate logistic regression was performed to identify any potential factors associated with very substantial weight gain from diagnosis to T3 assessment. The potential variables with *P* <  0.1 in univariate analysis were included into the multivariate logistic regression model. Similar analyses were performed to identify any potential factors associated with very substantial WHR increase from T0 to T3 assessment. All analyses were performed using SPSS 26.0; and *P* values < 0.05 at two-sided analysis were considered statistically significant.

## Results

### Patients’ characteristics

A total of 1462 patients who participated in this cohort were included into this analysis. The baseline demographic, clinical and lifestyle characteristics of patients are provided in Table 1. The mean age at diagnosis was 51.9 years (SD: 9.1). The median time from diagnosis to T0 assessment was 3.2 months. Overall, 84.1% of patients had education of high school or below, 71.1% were married, 32.4% had a monthly household income of more than 30, 000 HK dollars and 50.6% had full-time or part-time employment. At study entry, most of patients (61.6%) had no comorbidity and 53.5% of women were pre-menopausal. With regards to the clinical characteristics of breast tumor, majority of patients were staged as 0-II (80.4%), with tumor histology being invasive ductal carcinoma (83.8%) and ER positive (72.3%). The proportions of patients who received chemotherapy, radiotherapy and endocrine therapy were 75.2, 70.6 and 72.1%, respectively.

**Table 1:** Patients’ demographic, clinical and lifestyle characteristics collected at T0 assessment: the whole cohort and those who completed follow-up at T3

Characteristics	The whole cohort(*n* = 1462), n (%)	who completed follow-up at T3, (*n* = 1117), n (%)
Time from diagnosis to T0 assessment, median (range), months	3.2 (0.1–11.9)	3.2 (0.1–11.9)
Age at diagnosis, mean (SD), years	51.9 (9.1)	51.9 (8.8)
Age group at diagnosis, years		
< 40	150 (10.3)	111 (9.5)
40–49	468 (32.0)	383 (32.7)
50–59	552 (37.7)	444 (37.9)
≥60	292 (20.0)	233 (19.9)
Education level		
High school or below	1230 (84.1)	987 (84.3)
College or above	232 (15.9)	184 (15.7)
Marital status		
Married or cohabitation	1039 (71.1)	840 (71.7)
Unmarried or divorced or widowed	423 (28.9)	331 (28.3)
Family income, HKD/month		
< 15,000	683 (46.7)	530 (45.3)
15,000-30,000	452 (30.9)	375 (32.0)
30,000-50,000	204 (14.0)	174 (14.9)
≥50,000	123 (8.4)	92 (7.9)
Employment status		
Full time	545 (37.3)	433 (37.0)
Part time	195 (13.3)	146 (12.5)
Not working	722 (49.4)	592 (50.6)
Number of comorbidities		
0	901 (61.6)	726 (62.0)
1	371 (25.4)	296 (25.3)
≥2	190 (13.0)	149 (12.7)
Menopausal status at T0 assessment		
Pre-menopausal	782 (53.5)	627 (53.5)
Post-menopausal	680 (46.5)	544 (46.5)
Parity		
0	339 (23.2)	281 (24.0)
1	340 (23.3)	260 (22.2)
2	531 (36.3)	429 (36.6)
≥3	252 (17.2)	201 (17.2)
AJCC stage		
0-I	523 (35.8)	438 (37.4)
II	652 (44.6)	529 (45.2)
III	276 (18.9)	197 (16.8)
Missing	11 (0.8)	7 (0.6)
Histology		
IDC	1225 (83.8)	982 (83.8)
ILC	42 (2.9)	35 (3.0)
DCIS	94 (6.3)	70 (6.0)
Others	101 (6.9)	84 (7.2)
ER status, %		
Positive	1057 (72.3)	870 (74.3)
Negative	363 (24.8)	272 (23.2)
Missing	42 (2.9)	29 (2.5)
PR status, %		
Positive	810 (55.4)	675 (57.6)
Negative	605 (41.4)	463 (39.5)
Missing	47 (3.2)	33 (2.8)
HER 2 status, %		
Positive	381 (26.1)	305 (26.0)
Negative	966 (66.1)	786 (67.1)
Missing	115 (7.9)	80 (6.8)
Type of surgery		
Mastectomy	917 (62.7)	714 (61.0)
Conservation	545 (37.3)	457 (39.0)
Chemotherapy, %		
Yes	1100 (75.2)	875 (74.7)
No	362 (24.8)	296 (25.3)
Radiotherapy, %		
Yes	1032 (70.6)	825 (70.5)
No	430 (29.4)	346 (29.5)
Endocrine therapy, %		
Yes	1054 (72.1)	875 (74.7)
No	408 (27.9)	296 (25.2)
Height, median (range), cm	156 (137–177)	156 (137–177)
Weight, median (range), kg	56.0 (33.4–111.0)	55.4 (34.3–110.0)
BMI at diagnosis, kg/m^2^		
Underweight (< 18.5)	53 (3.6)	51 (4.4)
Normal (18.5–22.9)	713 (48.8)	540 (46.1)
Overweight (23–24.9)	297 (20.3)	251 (21.4)
Obese (≥25)	399 (27.3)	329 (28.1)
Waist circumference, median (range), cm	80.3 (58.5–126.5)	80.2 (58.5–126.5)
Hip circumference, median (range), cm	95.0 (78.0–136.5)	95.0 (78.0–135.0)
WHR at T0 assessment		
< 0.85	762 (52.1)	607 (51.8)
≥0.85	701 (47.9)	564 (48.2)
Sports participation 1-year before diagnosis		
Never	666 (45.6)	530 (45.3)
Rarely/occasionally	487 (33.1)	399 (34.1)
Frequently	309 (21.1)	242 (20.7)
Dietary energy intake 1-year before diagnosis, median (range), kcal/day	1620.3 (551.1–5787.3)	1616.6 (551.1–5787.3)
Dietary carbohydrate intake1-year before diagnosis, median (range), kcal/day	121.2 (56.9–191.2)	121.3 (56.9–191.2)
Dietary fat intake 1-year before diagnosis, median (range), g/1000 kcal/day	39.1 (14.2–62.7)	39.0 (14.2–62.7)
Coffee intake 1-year before diagnosis, ml/week		
< 200	991 (67.8)	785 (67.0)
≥200	471 (32.2)	386 (33.0)
Sugar-sweetened beverage intake1-year before diagnosis, ml/week		
< 200	1178 (80.6)	952 (81.3)
≥200	284 (19.4)	219 (18.7)
Vegetables and fruits intake 1-year before diagnosis, g/day		
< 400	496 (33.9)	399 (34.1)
≥400	966 (66.1)	772 (65.9)
Ever smoking before diagnosis		
Yes	22 (1.5)	21 (1.8)
No	1440 (98.5)	1150 (98.2)
Ever frequent alcohol intake before diagnosis (> 4 times/week)		
Yes	28 (1.9)	16 (1.4)
No	1434 (98.1)	1155 (98.6)

Abbreviations: *SD* standard deviation; *HKD* Hong Kong dollars; *BMI* body mass index; *AJCC* American joint Committee on cancer; *IDC* invasive ductal carcinoma; *ILC* invasive lobular carcinoma; *DCIS* ductal carcinoma in situ; *ER* estrogen receptor; *PR* progesterone receptor; *HER 2* human epidermal-growth-factor receptor 2; *MET* metabolic equivalent of task; *g* gram

﻿According to the BMI criteria for Asian population, 3.6% were underweight, 48.8% were normal weight, 20.3% were overweight and the remaining 27.3% were obese at breast cancer diagnosis. At study entry, the proportions of patients without or with abdominal obesity were 52.1 and 47.9%, respectively. With regards to lifestyle factors during the proceeding 1 year before breast cancer diagnosis, about half of patients (45.6%) never participated in sports activity, the median dietary energy intake was 1620.3 kcal/day, median carbohydrate intake was 121.2 g (g)/1000 kcal/day, and median fat intake was 39.1 g/1000 kcal/day. More than half of patients (66.1%) had vegetables and fruits ≥400 g/day. The proportions of patients who were ever smoker or ever frequent alcohol drinker were very small. The Characteristics of patients who completed follow-up at T3 were similar to the whole cohort. In addition, the characteristics of patients who were lost to follow-up at T1, T2, and T3 have been summarized in Supplementary Table 1.

### Distribution of patients’ BMI and weight change pattern from diagnosis to T0, T1, T2 and T3 assessment

**Fig. 1:**
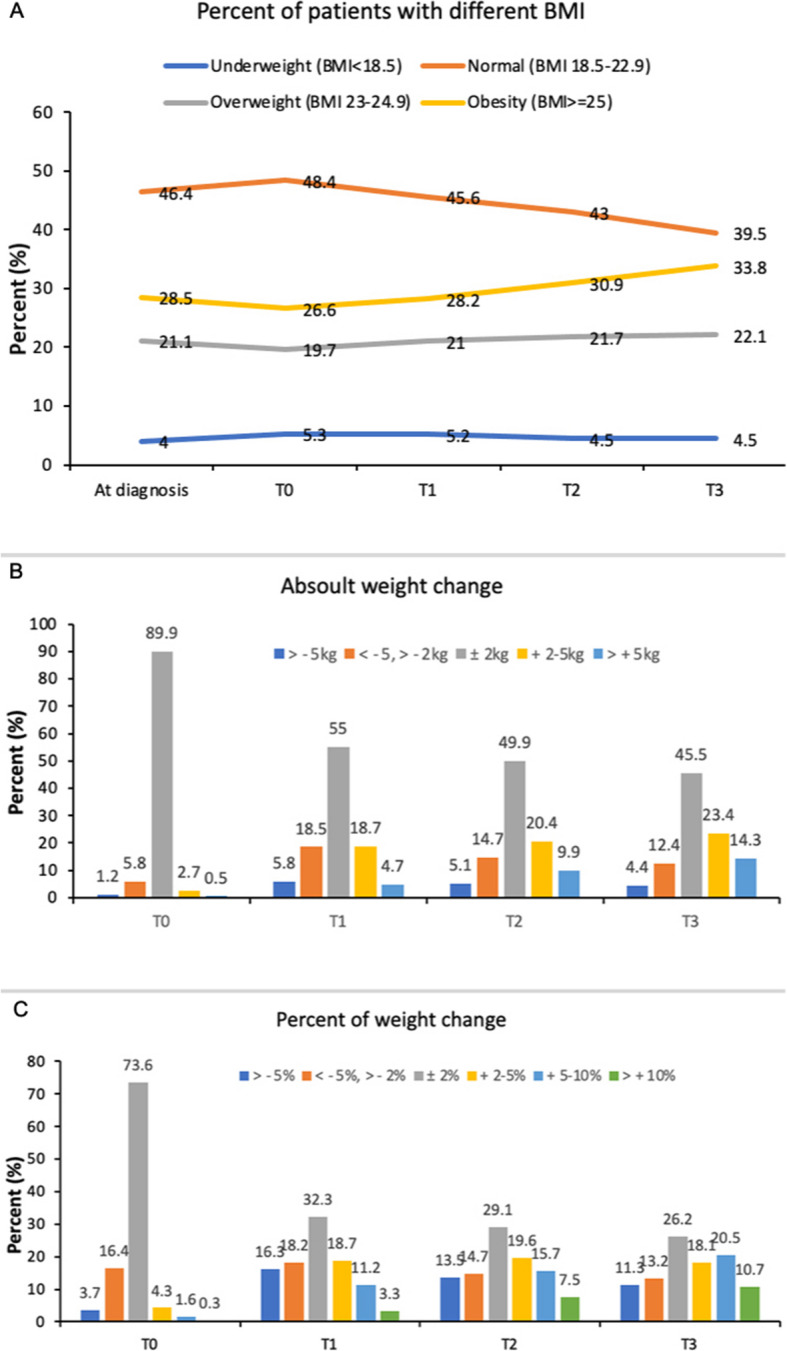
Distribution of patients by BMI or weight change. **A** Distribution of patients by BMI at diagnosis, T1, T2 and T3 assessment; **B**) Distribution of patients by absolute weight change categories from diagnosis to T0, T1, T2 and T3 assessment; **C**) Distribution of patients by percent of weight change categories from diagnosis to T0, T1, T2 and T3 assessment. Abbreviation: BMI, body mass index

The distribution of patients’ BMI at each time-point (from diagnosis to 60-months post-diagnosis) were summarized in Fig. 1A. The proportions of patients being underweight were relative stable, namely 4.0, 5.3, 5.2, 4.5 and 4.5% at diagnosis, T0, T1, T2 and T3 assessment; the proportions of patients being overweight were also stable during follow-ups; the corresponding figures were 21.1, 19.7, 21.0, 21.7 and 22.1% at diagnosis, T0, T1, T2 and T3 assessment, respectively. The proportions of patients with normal BMI slightly decreased in a progressive manner during follow-ups, namely 46.4, 48.4, 45.6, 43.0 and 39.5% at diagnosis, T0, T1, T2 and T3 assessment, respectively; while the proportion of patients with obesity slightly increased, namely 28.5, 26.6, 28.2, 30.9 and 33.8% at diagnosis, T0, T1, T2 and T3 assessment, respectively. In order to compare results from other countries, a sensitivity analysis was also undertaken using the WHO guideline for international use to define overweight and obesity (Supplementary Fig. 1) [36]. The proportions of patients being overweight were slightly increased during the first 5 years of survival; the corresponding figures were 22.3, 20.9, 21.2, 23.3 and 25.8% at diagnosis, T0, T1, T2 and T3 assessment, respectively. Overall, the proportions of patients being obese were low based on WHO guideline for international use and were stable during follow-ups, being 6.2, 5.8, 7.0, 7.6 and 8.0%.

Compared to weight at breast cancer diagnosis, the median weight change was − 0.5 kg, 0 kg, 0.5 kg, and 1 kg at T0, T1, T2 and T3, respectively. Absolute weight change from diagnosis to T0, T1, T2 and T3 were summarized in Fig. 1B. Most of the women had a relative stable weight (change within ±2 kg) at T0, T1, T2 and T3 assessment (89.9, 55, 49.9 and 45.5%, respectively) when compared to weight at diagnosis. The proportions of women who gained weight within 2-5 kg were 2.7, 18.7, 20.4 and 23.4% at T0, T1, T2 and T3, respectively; and the corresponding figures for patients who had weight gain of > 5 kg were 0.5, 4.7, 9.9 and 14.3%, respectively.

Percent of weight change from diagnosis to T0, T1, T2 and T3 were summarized in Fig. 1C. The percentage of women who gained weight by 2–5% were 4.3, 18.7, 19.6 and 18.1% at T0, T1, T2 and T3, respectively; the corresponding figures for weight gain 5–10% were 1.6, 11.2, 15.7 and 20.5% at T0, T1, T2 and T3 assessment, respectively; and the proportion of patients had weight gain > 10% were relatively low, 0.3, 3.3, 7.5 and 10.7% at T0, T1, T2 and T3 assessment, respectively.

### Category of WHR and change pattern from T0 to T1, T2 and T3 assessment

**Fig. 2:**
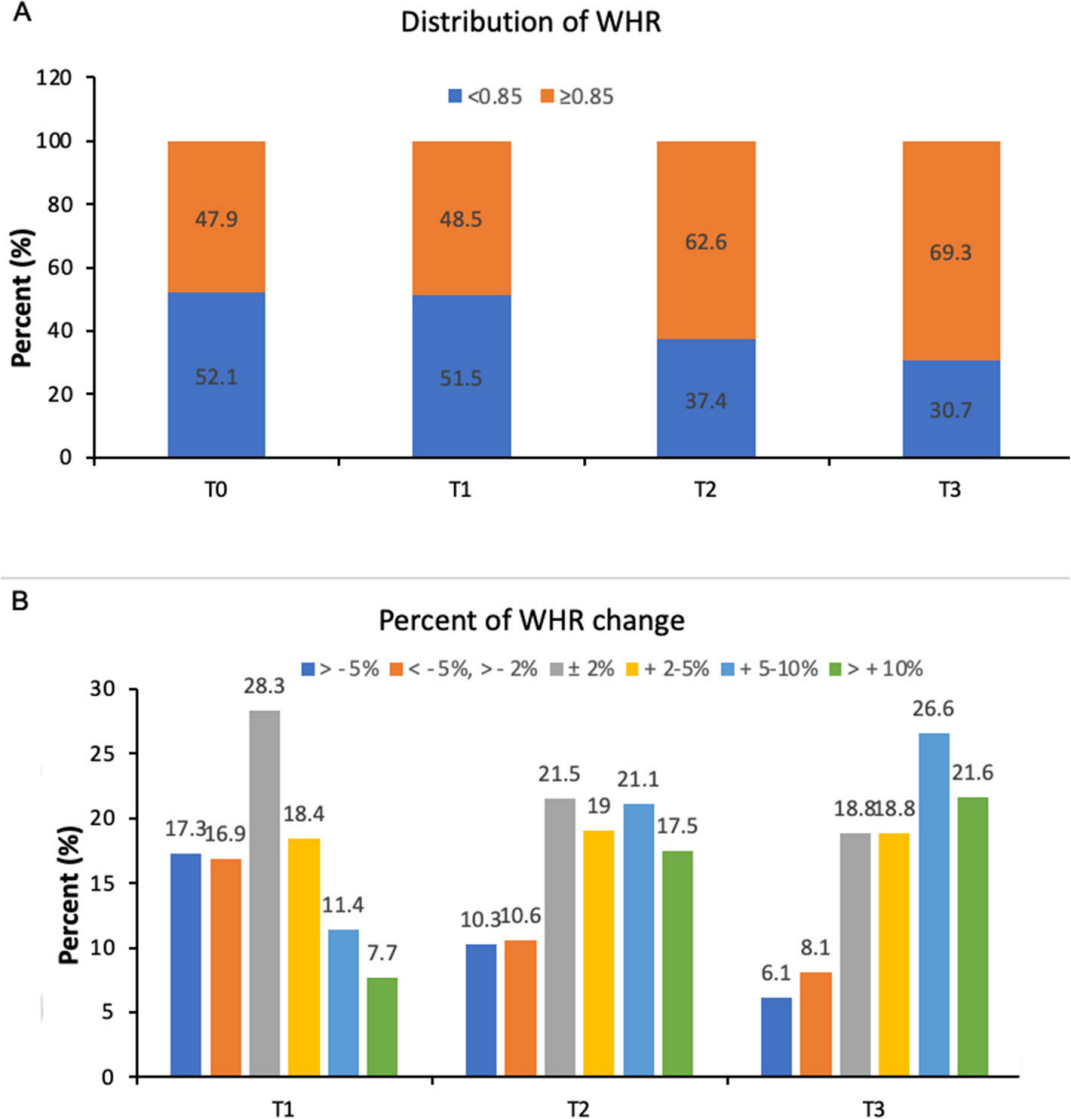
Distribution of patients by WHR. **A** Distribution of patients’ WHR categories at T0, T1, T2 and T3 assessment; **B**) Distribution of patients by percent of WHR change categories from T0 to T1, T2 and T3 assessment. Abbreviation: WHR, waist-to-hip ratio

The distribution of WHR categories at each follow-up were summarized in Fig. 2A.

The proportions of patients with WHR <  0.85 were 52.1, 51.5, 37.4 and 30.7% at T0, T1, T2 and T3 assessment, respectively.

With regards to percentage of WHR change, 28.3, 21.5 and 18.8% of patients had percentage of WHR change within ±2% from T0 to T1, T2 and T3 assessment, respectively (Fig. 2B). The percentage of women who increased WHR by 2–5% were 18.4, 19.0 and 18.8% at T1, T2 and T3, respectively; the corresponding figures for WHR increase 5–10% were 11.4, 21.1 and 26.6% at T1, T2 and T3, respectively; and the proportion of patients with WHR increase > 10% were 7.7, 17.5 and 21.6% at T1, T2 and T3, respectively. Overall, more patients had WHR increase during progressive follow-up.

### Analysis for risk factors associated with weight gain > 10% from diagnosis to T3 assessment

The outcomes of univariate and multivariate analyses on factors associated with very substantial weight gain (> 10%) were summarized in Table 2. Univariate analysis revealed that older age at breast cancer diagnosis (*P* = 0.001), had ≥2 comorbidities (*P* = 0.007), remained post-menopausal from T0 to T3 (*P* = 0.009), had ≥1 child-birth (*P* = 0.009), frequent sports participation from T1 to T3 assessment (*P* = 0.013), as well as average vegetables and fruits intake from T1 to T3 assessment ≥400 g/day (*P* = 0.001) were associated with less likelihood of weight gain. However, not working (*P* = 0.016) was associated with very substantial weight gain. ﻿On multivariate analysis, older age at breast cancer diagnosis [odds ratio (OR) for patients aged ≥60 years being 0.225, 95% confidence interval (CI): 0.073–0.697; *P* = 0.010) and frequent sports participation from T1 to T3 assessment (OR 0.523, 95%CI: 0.271–0.998; *P* = 0.049) were independent factors for less likelihood of weight gain.

**Table 2:** Univariate and multivariate analysis on factors associated with weight gain > 10% from diagnosis to T3 assessment, by stepwise logistic regression (*n* = 1171)

	Univariate analysis			Multivariate analysis		
	OR	95%CI for OR	P	OR	95%CI for OR	P
Age group at diagnosis			0.001			0.022
< 40	1	–	–	1	–	–
40–49	0.848	0.474–1.516	0.578	1.000	0.518–1.925	0.996
50–59	0.555	0.306–1.007	0.053	0.548	0.228–1.318	0.179
≥60	**0.235**	**0.104–0.528**	**< 0.001**	**0.225**	**0.073–0.697**	**0.010**
Education level						
High school or below	1	–				
College or above	1.314	0.817–2.115	0.260			
Marital status						
Married or cohabitation	1	–				
Unmarried or divorced or widowed	1.169	0.783–1.748	0.445			
Family income, HKD/month						
< 30,000	1	–				
≥30,000	1.193	0.778–1.828	0.419			
Employment status						
Working	1	–		1	–	
Not working	**1.590**	**1.088–2.323**	**0.016**	1.082	0.712–1.644	0.712
Number of comorbidities			0.026			0.160
0	1	–		1	–	
1	0.932	0.609–1.428	0.747	1.071	0.680–1.687	0.768
≥2	**0.312**	**0.134–0.727**	**0.007**	0.442	0.181–1.076	0.072
Menopausal status from T0 to T3 assessment			0.033			0.158
Pre-menopausal	1	–		1	–	
Peri-menopausal	0.688	0.421–1.124	0.135	0.865	0.492–1.522	0.616
Post-menopausal	**0.521**	**0.319–0.851**	**0.009**	1.585	0.716–3.507	0.256
Parity						
0	1	–		1	–	
≥1	**0.586**	**0.393–0.873**	**0.009**	0.771	0.499–1.919	0.242
AJCC stage			0.810			
0-I	1	–				
II	0.881	0.586–1.327	0.545			
III	0.993	0.583–1.691	0.980			
ER status, %						
Positive	1					
Negative	1.305	0.870–1.959	0.198			
PR status, %						
Positive	1					
Negative	1.042	0.716–1.515	0.831			
HER 2 status, %						
Positive	1					
Negative	0.932	0.614–1.416	0.743			
Type of surgery						
Mastectomy	1	–				
Conservation	0.898	0.612–1.319	0.584			
Chemotherapy, %						
No	1	–				
Yes	0.751	0.500–1.127	0.166			
Radiotherapy, %						
No	1					
Yes	0.844	0.568–1.255	0.403			
Endocrine therapy, %						
No	1					
Yes	0.852	0.562–1.290	0.448			
Average sports participation			0.009			0.052
from T1 to T3 assessment						
Never	1	–		1	–	
Rarely/occasionally	0.886	0.516–1.519	0.659	0.884	0.506–1.545	0.664
Frequently	**0.458**	**0.247–0.849**	**0.013**	**0.523**	**0.271–0.998**	**0.049**
Average dietary energy intake						
from T1 to T3 assessment						
≤median	1	–				
>median	1.057	0.729–1.532	0.769			
Average dietary carbohydrate intake from T1 to T3						
assessment, g/1000 kcal/day						
≤median	1	–				
>median	0.819	0.565–1.189	0.294			
Average dietary fat intake from T1 to T3 assessment, g/1000						
kcal/day						
≤median	1	–				
>median	1.266	0.872–1.837	0.216			
Average vegetables and fruits intake from T1 to T3 assessment, g/day						
< 400	1	–		1	–	
≥400	**0.568**	**0.365–0.886**	**0.013**	0.692	0.432–1.110	0.127
Average coffee intake from T1 to T3 assessment, ml/week						
< 200	1	–				
≥200	1.067	0.703–1.620	0.760			
Average sugar-sweetened beverage intake from T1 to T3 assessment, ml/week						
< 200						
≥200	1.644	0.957–2.825	0.072	1.201	0.678–2.124	0.530

Univariate logistic regression was performed to identify any potential factors associated with very substantial weight gain from diagnosis to T3 assessment. The potential variables with *P* < 0.1 in univariate analysis were included into the multivariate logistic regression model, including age group at diagnosis, employment status, number of comorbidities, menopausal status from T0 to T3 assessment, parity, average sports participation from T1 to T3 assessment and average sugar-sweetened beverage intake from T1 to T3 assessment

From T0 to T3 assessment, patients’ menopause status could be classified as three groups: pre-menopausal, peri-menopausal and post-menopausal. Peri-menopausal was defined as pre-menopausal patients at T0 who described a change in menopause status at T3

Abbreviations: *OR* odds ratio; *CI* confidence interval; *HKD* Hong Kong dollars; *AJCC* American joint Committee on cancer; *ER* estrogen receptor; *PR* progesterone receptor; *HER 2* human epidermal-growth-factor receptor 2; *MET* metabolic equivalent of task; *g* gram

### Analysis for risk factors associated with WHR increase > 10% from T0 to T3 assessment

Table 3 illustrates the outcomes of univariate and multivariate analyses on factors associated with WHR increase > 10% from T0 to T3 assessment. Univariate analysis revealed that older age at breast cancer diagnosis (patients aged 40–49 years, *P* = 0.024 patients aged ≥60 years, *P* = 0.016), had ≥2 comorbidities (*P* = 0.001) and ER negative (*P* = 0.019) were associated with less likelihood of very substantial WHR increase. On multivariate analysis, older age at breast cancer diagnosis (OR for patients aged 40–49 years 0.587, 95% CI: 0.361–0.956; *P* = 0.032), had ≥2 comorbidities (OR 0.417, 95% CI: 0.238–0.732; *P* = 0.002) and ER negative (OR 0.624, 95% CI: 0.440–0.886; *P* = 0.008) were all independent factors for very substantial WHR increase.

**Table 3:** Univariate and multivariate analysis on factors associated with WHR increase > 10% from diagnosis to T3 assessment, by stepwise logistic regression (n = 1171)

	Univariate analysis			Multivariate analysis		
	OR	95%CI for OR	P	OR	95%CI for OR	P
Age group at diagnosis, year			0.031			0.033
< 40	1	–	–	1	–	–
40–49	**0.572**	**0.352–0.927**	**0.024**	**0.587**	**0.361–0.956**	**0.032**
50–59	0.774	0.486–1.233	0.281	0.923	0.571–1.493	0.946
≥60	**0.520**	**0.305–0.886**	**0.016**	0.684	0.390–1.200	0.237
Education level			0.200			
High school or below	1	–				
College or above	1.272	0.881–1.836				
Marital status						
Married or cohabitation	1	–				
Unmarried or divorced or widowed	1.105	0.814–1.501	0.522			
Family income, HKD/month						
< 30,000	1	–				
≥30,000	1.184	0.855–1.638	0.310			
Employment status						
Working	1	–				
Not working	1.228	0.928–1.625	0.150			
Number of comorbidities			0.006			0.009
0	1	–		1	–	
1	0.878	0.633–1.217	0.434	0.859	0.610–1.210	0.385
≥2	**0.418**	**0.245–0.713**	**0.001**	**0.417**	**0.238–0.732**	**0.002**
Menopausal status from T0 to			0.445			
T3 assessment						
Pre-menopausal	1	–				
Peri-menopausal	1.146	0.760–1.727	0.515			
Post-menopausal	0.941	0.629–1.409	0.768			
Parity			0.259			
0	1	–				
≥1	0.832	0.605–1.145				
AJCC stage			0.203			
0-I	1	–				
II	0.840	0.620–1.136	0.258			
III	0.691	0.452–1.057	0.089			
ER status, %						
Positive	1			1		
Negative	**0.663**	**0.471–0.933**	**0.019**	**0.624**	**0.440–0.886**	**0.008**
PR status, %						
Positive	1					
Negative	0.895	0.674–1.189	0.444			
HER 2 status, %						
Positive	1					
Negative	1.124	0.814–1.554	0.478			
Type of surgery						
Mastectomy	1	–				
Conservation	1.236	0.931–1.640	0.143			
Chemotherapy, %						
No	1	–				
Yes	0.780	0.571–1.065	0.118			
Radiotherapy, %						
No	1					
Yes	1.081	0.794–1.472	0.621			
Endocrine therapy, %						
No	1	–				
Yes	1.229	0.882–1.712	0.223			
Average sports participation			0.655			
from T1 to T3 assessment						
Never	1	–				
Rarely/occasionally	0.843	0.543–1.311	0.449			
Frequently	0.942	0.595–1.491	0.799			
Average dietary energy intake from T1 to T3 assessment						
≤median	1	–				
>median	1.010	0.764–1.336	0.943			
Average dietary carbohydrate intake from T1 to T3						
assessment, g/1000 kcal/day						
≤median	1	–				
>median	1.052	0.796–1.391	0.722			
Average dietary fat intake from T1 to T3 assessment, g/1000						
kcal/day						
≤median	1	–				
>median	1.096	0.829–1.449	0.522			
Average vegetables and fruits intake from T1 to T3 assessment, g/day						
< 400	1	–				
≥400	0.792	0.550–1.140	0.210			
Average coffee intake from T1 to T3 assessment, ml/week						
< 200	1	–				
≥200	0.914	0.662–1.261	0.584			
Average sugar-sweetened beverage intake from T1 to T3						
assessment, ml/week						
< 200	1	–				
≥200	0.802	0.489–1.316	0.383			

Univariate logistic regression was performed to identify any potential factors associated with very substantial weight gain from diagnosis to T3 assessment. The potential variables with *P* < 0.1 in univariate analysis were included into the multivariate logistic regression model, including age group at diagnosis, number of comorbidities and ER status

From T0 to T3 assessment, patients’ menopause status could be classified as three groups: pre-menopausal, peri-menopausal and post-menopausal. Peri-menopausal was defined as pre-menopausal patients at T0 who described a change in menopause status at T3

Abbreviations: *WHR* waist-to-hip ratio; *OR* odds ratio; *CI* confidence interval; *HKD* Hong Kong dollars; *AJCC* American joint Committee on cancer; *ER* estrogen receptor; *PR* progesterone receptor; *HER 2* human epidermal-growth-factor receptor 2; *MET* metabolic equivalent of task; *g* gram

## Discussion

Based on a longitudinal breast cancer cohort, the present study prospectively measured anthropometric parameters during the first 5 years of survival. The results showed that weight gain was not common among Hong Kong women with breast cancer. Over the first 12-months and at 18-months post-diagnosis, the median weight change was − 0.5 kg and 0 kg, respectively. At 36-months to 60-months after diagnosis, it observed a modest weight gain with a medium value of 0.5 and 1 kg, respectively. At 60-months after diagnosis, only 14.3% of women had weight gain by > 5 kg; and the percentage of women who had weight gain by > 10% was 10.7%. Of note, nearly half of patients had abdominal obesity at study entry, and this figure gradually increased to nearly 70% at 60-months after diagnosis. Being older and having frequent sports participation were independent protective factors for very substantial weight gain in multivariate analysis. Additionally, aged 40–49 years at diagnosis, had ≥2 comorbidities and ER negative are independently associated with less likelihood of very substantial WHR increase.

The weight change pattern in the current study were inconsistent with findings from Western countries, which have generally reported weight gain following adjuvant treatment. Goodwin et al. reported the weight change from baseline to 1 year post-diagnosis among 535 women with newly diagnosed breast cancer in Canada, and showed that about 84% of women gained weight with a mean value of 1.6 kg [6]. Another study which included 185 women in United States (US) with early stage breast cancer, showed that the mean weight gain was 1.5 kg, 2.7 kg and 2.8 kg at 1 year, 2 year and 3 year after diagnosis, respectively [9]. Rock et al. examined the weight change from diagnosis to study enrollment (mean time since diagnosis: 26 months) among 1116 US breast cancer patients, showing that the mean weight gain was 2.7 kg and 60% of the participants reported weight gain [37]. In contrast to data from Western women, weight gain after breast cancer diagnosis among Asian women has been relatively modest. The Shanghai Breast Cancer Survival Study (SBCSS) reported that the median weight change from diagnosis to 6, 18 and 36 months post-diagnosis were 1.0, 2.0 and 1.0 kg, respectively; about 26, 37 and 33% of gained weight by ≥5% at 6, 18 and 36 months post-diagnosis, respectively [11]. It is noted that the magnitude of weight gain in the SBCSS was slightly greater than that obtained in the present study. Although the study design of the SBCSS was similar to the present cohort, the two studies enrolled patients diagnosed at different times; with the present study having enrolled patients who were diagnosed nearly 10 years later than the SBCSS (from 2011 to 2014). In a study of 260 Korean women with early stage breast cancer who received adjuvant treatment, the investigators reported that no weight gain was found after treatment, and the mean weight change was − 0.3 and − 0.4 kg at 1- and 2- year after treatment, respectively [10]. Another cross-sectional study included 280 premenopausal women (median age at diagnosis was 41 year) with breast cancer after chemotherapy in Hong Kong and reported similar weight change pattern: the median weight gain from diagnosis to 5 years after diagnosis was 1.8 kg, with 63.2% of women gaining weight by > 2% [13]. The varied results among studies in Asian women may be explained by difference in study design, variations in time interval for assessment since initial diagnosis, as well as diverse lifestyle habits.

Several previous studies have tried to investigate the associations between socio-demographic and clinical factors and weight change after diagnosis. In the Health, Eating, Activity and Lifestyle (HEAL) study, the results showed that postmenopausal women at diagnosis had greater weight gain than pre-menopausal women or women who had menopausal transition after diagnosis [8]. However, in the SBCSS, more weight gain was observed among women who were premenopausal during the first 6 months [11]. The present study supports the SBCSS findings that women who were pre-menopausal at study entry had higher risk of substantial weight gain than those who were postmenopausal in univariate analysis. Findings from studies in Western populations reported an association between chemotherapy and weight gain [6, 7, 9, 15]. However, this finding was not confirmed in Asian population. For example, the Korean study and the SBCSS did not find a weight gain after adjuvant chemotherapy [10, 11]. Similarly, the present study did not show chemotherapy to be associated with the risk of very substantial weight gain. On the other hand, the present study found that older age was associated with reduced risk of weight gain even in multivariate analysis, which was supported by two previous studies in Chinese women with breast cancer [11, 13].

Several studies have reported a significant decrease in physical activity during and after treatment, and this lifestyle change may be another reason for post-diagnosis weight gain [7, 38, 39]. Chen et al. explored the potential predictors of weight change in SBCSS and reported that higher exercise level was marginally related to weight loss [16]. Similar relationship was observed in the current study, higher level of physical activity was statistically associated with lower risk of very substantial weight gain in both univariate and multivariate analysis. Those findings suggested that high level of physical activity might prevent post-diagnosis weight gain. The results from SBCSS suggested that higher energy intake was related to greater weight gain [16]; while findings from the present study and Yaw et al’ s study showed that total energy intake was not associated with weight change [12].

A number of reports have investigated the relationship between post-diagnosis weight gain and breast cancer prognosis, and strongly supported that post-diagnosis weight gain was related to higher risk of mortality among breast cancer survivors. In 2015, Playdon et al. systematically summarized these data, including 12 studies and a total of 23,832 breast cancer patients [40]. The meta-analysis showed that patients who gained weight by ≥5% was associated with increased risk of overall mortality compared with patients who maintained their body weight (defined as weight change < ±5%) [40]. However, it should be noted that evidence from Chinese women with breast cancer has been limited, with only one study showing that women who gained ≥5 kg had higher mortality than those who maintained their weight [41].

WHR was regarded as an index for the measurement of ﻿central adiposity [35]. In general population, ﻿abdominal adipose tissue (which is positively associated with waist circumference and waist-hip ratio) is associated with a range of metabolic abnormalities, including ﻿decreased glucose tolerance, reduced insulin sensitivity and adverse lipid profiles [35]. The present study found that nearly half of women with breast cancer had central adiposity and this phenomenon is more severe as survival time increased. To our knowledge, this is the first study to describe the change pattern of WHR. Multivariate analysis showed that patients aged 40–49 years and had ≥2 comorbidities were less likely to had very substantial WHR increase when compared to those aged < 40 years and those had no comorbidity, respectively; suggesting that those patients may be more cared about abdominal obesity. Moreover, the present study suggested that patients with ER-negative disease was associated with less likelihood of very substantial WHR increase when compared to patients with ER positive; this may be related to use of adjuvant endocrine therapy, in particular, tamoxifen.

Of interests, a few studies have investigated whether central adiposity status after breast cancer diagnosis was associated with detrimental outcomes of breast cancer [42–45]. For a given BMI, it has been widely reported that Asians tend to have a higher fat percentage and a higher proportion of abdominal adiposity than western populations, [46, 47] it highlights the need to address this issue in specific ethnic groups.

This study was based on data from a longitudinal cohort study with a large sample size, with quantitatively compared the changes of body weight and WHR from immediately post-diagnosis to 5 years of survival. However, a few limitations should be noted. First, ﻿the change pattern was not compared with cancer-free women of similar age; it is, therefore, unclear whether these observed changes can be attributed to aging perse or to breast cancer and its treatment. Second, the baseline measurements of WHR were conducted within 1 year after breast cancer diagnosis; with no data captured right after diagnosis. However, the interval between diagnosis to study entry was relatively short (with median time of 3.2 months), significant WHR changes would not be expected. Third, the multivariate analysis for potential predictive factors could only be regarded as an exploratory analysis given that no clear hypothesis had been stated at priori. Fourth, as patients may experience weight loss after cancer recurrence or before death, and those patients were not included in the present analyses. As such, excluding these women who have cancer recurrence or died during the first 5 years of survival may lead to including more women with weight gain. Fifth, as the original study primarily aimed to investigate the associations between lifestyle factors with breast cancer recurrence and mortality, data on body fat was not collected during the assessments. As such, this report is unable to describe the patterns of body fat change. Finally, as the survival data in the present cohort was not mature enough, this study did not investigate the association between weight gain and breast cancer outcomes. Prospective follow-up is needed to further explore such association in Chinese women with breast cancer.

## Conclusion

Based on a longitudinal observational cohort of Chinese female patients with early-stage breast cancer in Hong Kong, this study compared the change pattern of body weight and WHR during the first 5 years of survival, and explored the potential factors related to very substantial changes. The study identifies that breast cancer patients in Hong Kong experienced a modest weight gain over the first 5 years of survival, with only about 10% of women gained weight by > 10%. WHR analysis found that nearly half of patients had central adiposity at breast cancer diagnosis, but the proportion increased to nearly 70% at 60-months follow-up. Multivariate analyses indicated that frequent sports participation during the first 5 years of survival was significantly associated with lower risk of great weight gain after diagnosis. ﻿Weight management in breast cancer survivorship is an essential component and should be integrated into the survivorship care. Furthermore, central adiposity has become a contemporary public health issue especially for Asians, and the incorporation of healthy abdominal circumference education and management has the potential to improve the length and quality of cancer survivorship.

## Supplementary Information

**Additional file 1; Supplementary Table 1**. The demographic, clinical and lifestyle characteristics collected at T0 assessment among patients who were loss to follow-up at T1, T2, and T3. **Supplementary Fig. 1** Distribution of patients’ BMI at diagnosis, T1, T2 and T3 assessment using the WHO guideline for international use. Abbreviation: BMI, body mass index; WHO, World Health Organization

## Data Availability

All analyzed data during the current study were presented in the main manuscript. The original datasets are available from the corresponding author on reasonable request.

## Abbreviations

WHR: Waist-to-hip ratio
ACS: American cancer society
ASCO: American society of clinical oncology
QoL: Quality of life
HKNKBCSS: The Hong Kong NTEC-KWC breast cancer survival study
AJCC: American joint committee on cancer
ER: Estrogen receptors
PR: Progesterone receptors
HER2: Human epidermal-growth-factor receptor 2
BMI: Body mass index
OR: Odds ratio
SBCSS: Shanghai breast cancer survival study
