# Supplementary material for: Weight and waist-to-hip ratio change pattern during the first five years of survival: data from a longitudinal observational Chinese breast cancer cohort
Source: BMC Cancer. 2021 Jul 20;21:839. doi: 10.1186/s12885-021-08554-5 (PMC8293589; doi:10.1186/s12885-021-08554-5)
Supplement: Supplementary file 1 — Additional file 1; Supplementary Table 1. The demographic, clinical and lifestyle characteristics collected at T0 assessment among patients who were loss to follow-up at T1, T2, and T3. Supplementary Fig. 1 Distribution of patients’ BMI at diagnosis, T1, T2 and T3 assessment using the WHO guideline for international use. Abbreviation: BMI, body mass index; WHO, World Health Organization [file 12885_2021_8554_MOESM1_ESM.docx]

Supplementary materials

Supplementary Table 1. The demographic, clinical and lifestyle characteristics collected at T0 assessment among patients who were loss to follow-up at T1, T2, and T3

| Characteristics | Patients who had loss follow-up at T1 (n=152) | Patients who had loss follow-up at T2 (n=300) | Patients who had loss follow-up at T3 (n=291) |
| --- | --- | --- | --- |
|  | Number of patients and percentage (n, %) | Number of patients and percentage (n, %) | Number of patients and percentage (n, %) |
| Time from diagnosis to T0 assessment, median (range), months | 3.1 (0.7-11.5) | 3.2 (0.5-11.8) | 3.3 (0.7-11.8) |
| Age at diagnosis, mean (SD), years | 49.8 (10.3) | 51.0 (10.2) | 51.6 (10.2) |
| Age group at diagnosis, years  <40  40-49  50-59  ≥60 | 25 (16.4)  54 (35.5)  47 (30.9)  26 (17.1) | 43 (14.3)  97 (32.3)  103 (34.3)  57 (19.0) | 39 (13.4)  85 (29.2)  108 (37.2)  59 (20.2) |
| Education level  High school or below  College or above | 118 (77.6)  34 (22.4) | 242 (80.7)  58 (19.3) | 243 (83.5)  48 (16.5) |
| Marital status  Married or cohabitation  Unmarried or divorced or widowed | 109 (71.7)  43 (28.3) | 204 (68.0)  96 (32.0) | 199 (68.4)  92 (31.6) |
| Family income, HKD/month  <15,000  15,000-30,000  30,000-50,000  ≥50,000 | 73 (48.0)  43 (28.3)  20 (13.2)  16 (10.5) | 146 (48.7)  87 (29.0)  34 (11.3)  33 (11.0) | 153 (52.6)  77 (26.5)  30 (10.3)  31 (10.7) |
| Employment status  Full time  Part time  Not working | 56 (36.8)  14 (9.2)  82 (53.9) | 109 (36.3)  46 (15.3)  145 (48.3) | 112 (38.5)  49 (16.8)  130 (44.7) |
| Number of comorbidities  0  1  ≥2 | 101 (66.4)  31 (20.4)  20 (13.2) | 185 (61.7)  76 (25.3)  39 (13.0) | 175 (60.1)  75 (25.8)  41 (14.1) |
| Menopausal status at T0 assessment  Pre-menopausal  Post-menopausal | 98 (64.5)  54 (35.5) | 166 (55.3)  134 (44.7) | 155 (53.3)  136 (46.7) |
| Parity  0  1  2  ≥3 | 41 (27.0)  39 (25.7)  43 (28.3)  29 (19.1) | 63 (21.0)  77 (25.7)  111 (37.0)  49 (16.3) | 58 (19.9)  80 (27.5)  102 (35.1)  51 (17.5) |
| AJCC stage  0-I  II  III  Missing | 57 (37.5)  58 (38.2)  32 (21.1)  5 (3.3) | 105 (35.0)  112 (37.3)  77 (25.7)  6 (2.0) | 85 (29.2)  123 (42.3)  79 (27.1)  4 (1.4) |
| Histology  IDC  ILC  DCIS  Others | 119 (78.3)  4 (2.6)  17 (11.2)  12 (7.9) | 248 (82.6)  8 (2.7)  23 (7.7)  21 (7.0) | 245 (84.1)  7 (2.4)  22 (7.6)  17 (5.8) |
| ER status, %  Positive  Negative  Missing | 93 (61.2)  43 (28.3)  16 (10.5) | 209 (69.7)  76 (25.3)  15 (5.0) | 187 (64.3)  91 (31.3)  13 (4.5) |
| PR status, %  Positive  Negative  Missing | 66 (43.4)  69 (45.4)  17 (11.2) | 157 (52.3)  127 (42.3)  16 (5.3) | 135 (46.4)  180 (61.9)  35 (12.1) |
| HER 2 status, %  Positive  Negative  Missing | 35 (23.0)  86 (56.6)  31 (20.4) | 66 (22.0)  193 (64.3)  41 (13.6) | 76 (26.1)  86 (56.6)  31 (20.4) |
| Type of surgery  Mastectomy  Conservation | 96 (63.1)  56 (36.8) | 197 (65.7)  103 (34.3) | 203 (69.7)  88 (30.2) |
| Chemotherapy, %  Yes  No | 99 (65.1)  53 (34.9) | 208 (69.3)  92 (30.7) | 225 (77.3)  66 (22.7) |
| Radiotherapy, %  Yes  No | 100 (65.8)  52 (34.2) | 209 (69.7)  91 (30.3) | 207 (71.1)  84 (28.9) |
| Endocrine therapy, %  Yes  No | 84 (55.3)  68 (44.7) | 194 (64.7)  106 (35.4) | 179 (61.5)  112 (38.5) |
| Height, median (range), cm | 157 (141-171) | 157 (139-171) | 157 (141-174) |
| Weight, median (range), kg | 56.1 (40.6-95.0) | 56.8 (33.4-95.0) | 57.0 (37.7-95.0) |
| BMI at diagnosis, kg/m^2^  Underweight (<18.5)  Normal (18.5-22.9)  Overweight (23-24.9)  Obese (≥25) | 11 (7.2)  75 (49.3)  26 (17.1)  40 (26.3) | 12 (4.0)  139 (46.3)  64 (21.3)  85 (28.3) | 8 (2.7)  139 (47.7)  57 (19.6)  87 (29.9) |
| Waist circumference, median (range), cm | 80.6 (61.7-114.5) | 80.2 (58.5-114.5) | 80.8 (58.5-114.5) |
| Hip circumference, median (range), cm | 95.7 (78.0-136.5) | 95.1 (78.5-136.5) | 95.1 (78.5-136.5) |
| WHR at T0 assessment  < 0.85  ≥0.85 | 89 (58.7)  63 (41.3) | 165 (54.8)  135 (45.2) | 89 (58.7)  63 (41.3) |
| Sports participation 1-year before diagnosis  Never  Rarely/occasionally  Frequently | 75 (49.3)  48 (31.6)  29 (19.1) | 127 (42.3)  104 (34.7)  69 (23.0) | 136 (46.7)  88 (30.2)  67 (23.0) |
| Dietary energy intake 1-year before diagnosis, median (range), kcal/day | 1623.4  (704.6-4864.8) | 1658.9  (652.3-4864.8) | 1655.3  (652.3-4421.0) |
| Dietary fat intake, median (range), g/1000 kcal/day | 39.6 (17.1-59.7) | 39.5 (17.1-62.5) | 39.5 (17.1-62.5) |
| Coffee intake 1-year before diagnosis, ml/week  <200  ≥200 | 112 (73.7)  40 (26.3) | 217 (72.3)  83 (27.7) | 206 (70.8)  85 (29.2) |
| Sugar-sweetened beverage intake1-year before diagnosis, ml/week  <200  ≥200 | 120 (78.9)  32 (21.2) | 236 (78.7)  64 (21.3) | 226 (77.7)  65 (22.3) |
| Vegetables and fruits intake 1-year before diagnosis, g/day  <400  ≥400 | 55 (36.2)  97 (63.8) | 86 (28.7)  214 (71.3) | 97 (33.3)  194 (66.7) |
| Ever smoking before diagnosis  Yes  No | 6 (3.9)  146 (96.1) | 5 (1.7)  295 (98.3) | 6 (2.1)  285 (97.9) |
| Ever frequent alcohol intake before diagnosis (> 4 times/week)  Yes  No | 8 (5.3)  144 (94.7) | 5 (1.7)  295 (98.3) | 7 (2.4)  284 (97.6) |

Abbreviations: SD, standard deviation; HKD, Hong Kong dollars; BMI, body mass index; AJCC, American joint Committee on cancer; IDC, invasive ductal carcinoma; ILC, invasive lobular carcinoma; DCIS, ductal carcinoma in situ; ER, estrogen receptor; PR, progesterone receptor; HER 2, human epidermal-growth-factor receptor 2; MET, metabolic equivalent of task; g, gram.


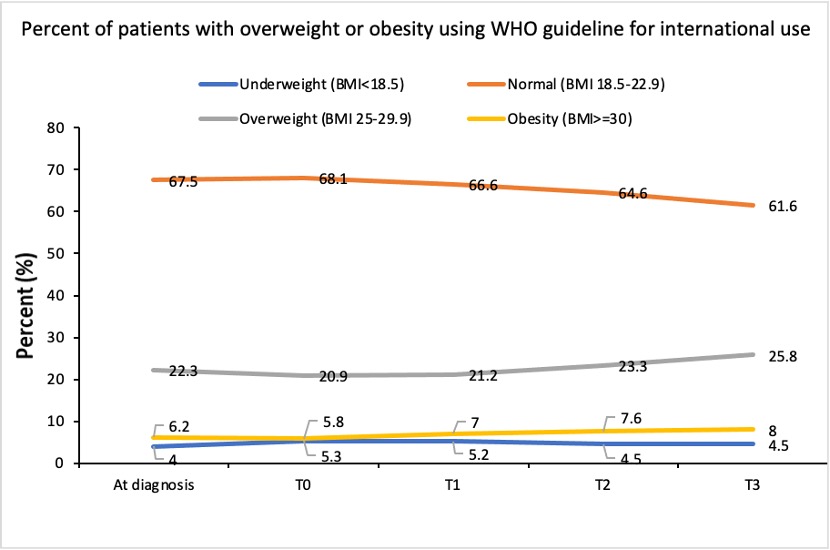
Supplementary Figure 1. Distribution of patients’ BMI at diagnosis, T1, T2 and T3 assessment using the WHO guideline for international use

Abbreviation: BMI, body mass index; WHO, World Health Organization
